# A Review of Therapeutic Drug Monitoring in Patients with Inflammatory Bowel Disease Receiving Combination Therapy

**DOI:** 10.3390/jcm12206577

**Published:** 2023-10-17

**Authors:** Sanket Patel, Andres J. Yarur

**Affiliations:** 1 Virtua Health, Voorhees, NJ 08043, USA; spatel11@virtua.org; 2 Cedars-Sinai Medical Center, 8730 Alden Dr., Los Angeles, CA 90048, USA

**Keywords:** inflammatory bowel disease, ulcerative colitis, Crohn’s disease, therapeutic drug monitoring, combination therapy, biologics, immunomodulators

## Abstract

**Background**: Inflammatory Bowel Disease (IBD) impacts millions worldwide, presenting a major challenge to healthcare providers and patients. The advent of biologic therapies has enhanced the prognosis, but many patients exhibit primary or secondary non-response, underscoring the need for rigorous monitoring and therapy optimization to improve outcomes. **Objective**: This narrative review seeks to understand the role of therapeutic drug monitoring (TDM) in optimizing treatment for IBD patients, especially for those on combination therapies of biologics and immunomodulators. **Methods**: A comprehensive synthesis of the current literature was undertaken, focusing on the application, benefits, limitations, and future directions of TDM in patients receiving a combination of biologic therapies and immunomodulators. **Results**: While biological therapies have improved outcomes, rigorous monitoring and therapy optimization are needed. TDM has emerged as a pivotal strategy, enhancing outcomes cost-effectively while reducing adverse events. While most data pertain to monotherapies, TDM’s applicability also extends to combination therapy. **Conclusion**: TDM plays a crucial role in the treatment optimization of IBD patients on combination therapies. Further research is needed to fully understand its potential and limitations in the broader context of IBD management.

## 1. Introduction

Inflammatory Bowel Disease (IBD) is a chronic condition of the gastrointestinal tract, classically differentiated into Crohn’s disease (CD) or ulcerative colitis (UC) based on the location, behavior, and histopathologic characteristics. The disease can manifest in periods of flare-ups and remission, potentially leading to severe complications, diminished quality of life, and increased mortality if not adequately managed. Optimal management strategies are essential to alleviate symptoms, prevent complications, and enhance long-term prognosis, ultimately aiming to improve patients’ quality of life.

A growing number of options are available in the therapeutic armamentarium of IBD, with the first biologic being approved more than twenty years ago [1]. While infliximab was the first anti-tumor necrosis factor (TNF) agent approved, several other anti-TNF therapies have since become available for use in IBD, such as adalimumab, certolizumab, and golimumab. Vedolizumab, ustekinumab, and risankizumab are newer biologics with an alternative mechanism of action that have been shown to be effective and have been approved for Crohn’s disease (CD) and/or ulcerative colitis (UC). Even though these are effective drugs at inducing and maintaining remission in IBD, a high number of patients do not respond or develop a loss of response over time [1,2,3,4,5,6,7,8,9,10,11].

Given the need for effective strategies in this complex therapeutic landscape, combination therapy with biologics and immunomodulators is commonly employed. Within this context, therapeutic drug monitoring (TDM) has emerged as a valuable tool to optimize treatment, and while its use in combination therapy can be beneficial, there are nuances and limitations that warrant a closer look. In general, TDM aims to recapture the response to biologic therapy or prevent the loss of response. It involves measuring drug concentrations and anti-drug antibodies (ADAs) in specific clinical settings. In patients with a partial or loss of response to anti-TNF therapy, it allows for the identification of those who may benefit from dose adjustment as opposed to those who are likely to benefit from a switch to another agent with either the same or an alternative mechanism of action. Importantly, TDM can effectively guide the use and optimization of combination therapies with immunomodulators like thiopurines and methotrexate [12]. Patients needing combination therapy usually have a higher disease burden and more aggressive phenotype; hence, the optimization of treatment regimens in this population becomes even more important. In addition to the association between improved clinical outcomes and higher drug concentrations, cost-effectiveness has been demonstrated through this approach [13,14].

Overall, TDM has gained traction as a potential tool to tailor treatment regimens, minimize adverse effects, and cost-effectively improve drug efficacy. This review aims to provide an overview of TDM’s role in patients with IBD receiving combination therapy, highlighting available evidence and discussing practical aspects, benefits, limitations, and future directions.

## 2. Methodology

**Figure 1:**
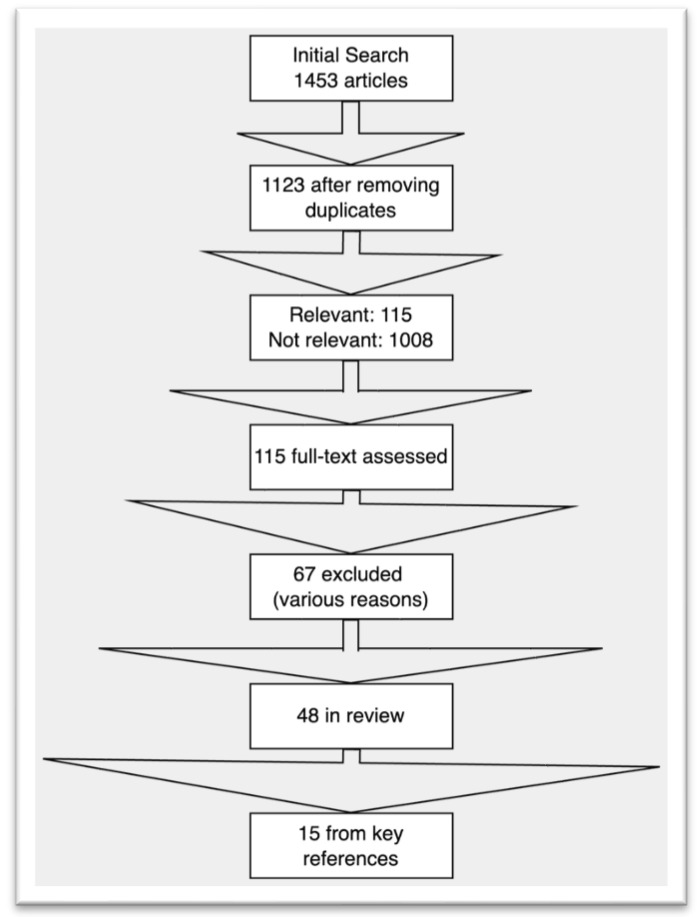
Flowchart of selection of review articles.

Our search strategy involved identifying relevant studies on TDM in IBD patients treated with combination therapy. Electronic databases, including PubMed, MEDLINE, and EMBASE, were queried for the appropriate studies published up to 2023. Keywords such as “ulcerative colitis”, “Crohn’s disease”, “inflammatory bowel disease”, “therapeutic drug monitoring”, “combination therapy”, “biologics”, and “immunomodulators” were applied to identify relevant studies. We included studies discussing TDM in the context of IBD treatment with combination therapy, studies examining the impact of TDM on clinical outcomes, studies presenting data on drug levels of biologics concerning treatment outcomes, and studies available in the English language (Figure 1). Studies without direct relevance to the topic were excluded. To ensure the integrity of our review and minimize bias, two independent reviewers screened titles and abstracts. The full-text articles were reviewed based on inclusion criteria to identify the appropriate studies; any disagreements were resolved through discussion, and no automation tools were employed during the screening and inclusion process. Our review considered various factors, including participants’ characteristics; study design; TDM methodologies; clinical outcomes; key findings; and intervention details, such as the type of therapy, dosage, durations, levels, and specific combinations. Our findings are presented in a narrative synthesis, highlighting the variations and trends observed in the literature regarding TDM in IBD combination therapy.

## 3. Therapeutic Drug Monitoring Strategies in Inflammatory Bowel Disease

The observations that led to TDM in IBD as a strategy were paved by an era of monotherapy thiopurine use as the capabilities to measure its sub-metabolites in red blood cells emerged [15]. Thiopurines are available as pro-drugs that undergo a series of enzymatic pathways leading to the production of several sub-metabolites. Among those, two have been found to have clinical relevance and are used to monitor and optimize the clinical care of patients receiving thiopurines. Of the two, higher 6-thioguanine nucleotide (6-TGN) concentrations have been associated with higher rates of efficacy but also a higher risk of myelotoxicity, while higher 6-methylmercaptopurine nucleotide (6-MMP) concentrations are linked with an increased risk of developing hepatotoxicity [16,17]. Due to its low cost, thiopurine monotherapy can still play an important role in the treatment of IBD, even though it is currently mainly used in the setting of combination therapy with biologics [16,18,19,20].

The use of TDM In IBD really surged with the wide use of anti-TNF and the relatively high rates of primary and secondary non-response seen with these drugs. Observational studies have found a clear association between higher serum concentrations of anti-TNFs and better outcomes, such as clinical remission, biochemical response, and endoscopic improvement [21,22,23]. Higher anti-TNF concentrations are also associated with histologic remission [24]. Despite this dose–response relationship, increasing exposure to the drug (e.g., increasing the dose) does not always translate into a better response, highlighting the complex pharmacokinetic and pharmacodynamic mechanisms of these agents and IBD [25,26]. Conversely, the development of ADAs is associated with a higher drug clearance, a loss of response, and the development of adverse events.

**Figure 2:**
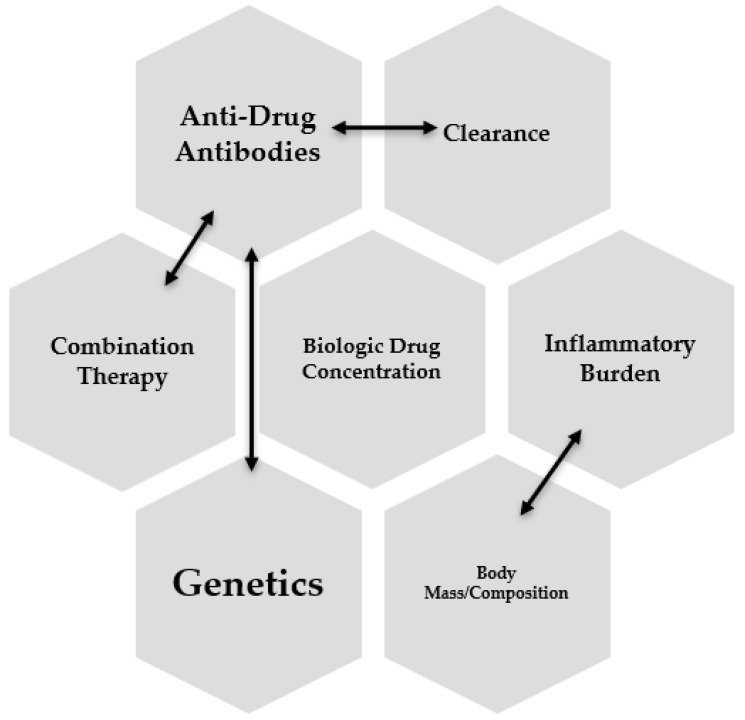
Interconnected factors influencing biologic drug levels.

Several interconnected factors potentially influence the pharmacokinetics of anti-TNF. Inflammatory burden, obesity, genetics, immunogenicity, and the concomitant use of immunomodulators are all associated with anti-TNF pharmacokinetics (Figure 2).

Two strategies for applying TDM in IBD patients receiving anti-TNFs are currently well recognized. Reactive TDM involves measuring drug levels and ADAs in those experiencing non-response to therapy. Patients with low drug levels and no significant immunogenicity are more likely to benefit from dose escalation, while those with either high drug levels or the presence of high ADA titers are more likely to profit from a change in therapy.

Proactive TDM involves monitoring drug levels irrespective of the response to therapy while optimizing the dosage to maintain an “optimal” drug level. The main goal of proactive TDM is to maintain optimal exposure to the drug, avoiding under-exposure and the development of immunogenicity, ultimately preventing the loss of response [21,22,24,27]. Even though this strategy should theoretically offer significant advantages, the evidence is conflicting. Table 1 shows select studies comparing proactive and reactive monitoring. 

Both the TAXIT (Trough Concentration Adapted Infliximab Treatment) and TAILORIX (Tailored Treatment With Infliximab for Active Crohn’s Disease) randomized trials looked into the effectiveness of proactive TDM in patients receiving infliximab for IBD [28,29,30]. Even though the overall conclusion of these studies was that proactive TDM is not better than as-needed dose adjustment, multiple factors need to be considered when interpreting the results of these studies and applying them to clinical practice. For example, there is significant heterogenicity in phenotypes (acute severe UC or perianal CD, for example), drug clearance, and other important variables among patients. Considering these differences, target infliximab levels are likely heterogeneous among patients. Factors such as body composition (particularly with higher visceral adipose tissue) and an elevated inflammatory burden may influence these goal concentrations [12,34,35,36]. Proactive TDM has also been studied in patients receiving adalimumab. PAILOT (Pediatric Crohn’s Disease Adalimumab Level-based Optimization Treatment) was a non-blinded randomized controlled trial of pediatric patients with CD on adalimumab, and it demonstrated that proactive dose adjustment was more efficacious than a reactive approach when looking into sustained corticosteroid-free clinical remission [31]. While a large Norwegian study (NOR-DRUM A and B) showed no benefit during the induction phase, it did demonstrate the advantage of proactive TDM during the maintenance phase across IBD and other immune-mediated diseases, suggesting that the timing of proactive TDM application may be important [32,33].

TDM is a more cost-effective approach than empiric treatment adjustments. Velayos and colleagues compared the monitoring strategy and empiric drug escalation and found that TDM was more effective [37]. A randomized control trial in Denmark found that TDM-based treatment was more economical without a difference in clinical efficacy [38]. Proactive TDM is also slightly more cost-effective than the reactive approach, while TDM for thiopurines is significantly cost-effective [39,40].

Another strategy that could also promise a potential benefit is precision dosing, offering a more personalized approach to management. A Scandinavian study randomized a group of CD and UC patients in remission on infliximab to continue the drug with a dose based on a pharmacokinetic-modeled dashboard system to target trough levels ≥ 3 µg/mL or to continue standard dosing. After a year of follow-up, patients dosed based on the dashboard had higher rates of sustained clinical remission than those who continued standard therapy [41]. Even though the current evidence is conflicting, proactive TDM could potentially evolve into a personalized approach that considers individual patient factors, such as disease severity, comorbidities, pharmacokinetic variability, and phenotypic and genetic characteristics, to tailor treatment regimens to each patient. Overall, while the evidence is still equivocal, proactive TDM can be recommended, especially for patients receiving monotherapy or those with a high drug clearance, such as acute severe UC. Proactive TDM can also predict the development of immunogenicity and non-response when re-initiating infliximab after a drug holiday [42,43].

The role of TDM in biologics other than infliximab and adalimumab is less established. Most observational studies have shown a relationship between exposure and response [44,45,46,47,48,49]. Despite this, there is a lack of clinical evidence showing that dose escalation improves outcomes. The ENTERPRET study was an open-label, randomized trial that included patients with UC starting vedolizumab, who had drug clearance and non-response at week six. Patients were randomized to either continue the standard maintenance treatment (300 mg every 8 weeks) or receive a higher dose regimen (600 mg followed by 300 mg or 600 mg every 4 weeks (based on drug clearance)). After 30 weeks, there were no differences in clinical or endoscopic outcomes between the patients who were randomized to receive higher doses and those who continued standard treatment [50]. The POWER study was a double-blind, randomized, controlled trial that assessed CD patients with secondary non-response to ustekinumab who underwent dose intensification with IV re-induction or placebo followed by subcutaneous ustekinumab maintenance therapy. While inflammatory biomarkers and endoscopic outcomes did improve, the primary endpoint of clinical response at week 16 was not achieved [51]. Another important variable that distinguishes the use of TDM in anti-TNF and vedolizumab or ustekinumab is the very low rate of immunogenicity seen with these newer biologics [9,10,11,52].

As opposed to ustekinumab and vedolizumab, immunogenicity is commonly seen with most anti-TNF agents. ADAs can lead to low drug levels, adverse reactions, and worse outcomes [12,53]. Immunogenicity occurs in up to 65% of infliximab-treated patients, 38% of adalimumab-treated patients, 25% of certolizumab-treated patients, and 3% of golimumab-treated patients, according to a large meta-analysis [54]. Studies suggest that the risk of immunogenicity can be reduced and potentially be reversible by using anti-TNF in combination with methotrexate or thiopurines, effectively decreasing antibody formation and increasing the effective drug concentration [12,13,55,56,57,58,59,60].

## 4. Evidence for TDM in Combination Therapy

Combination therapy can offer improved clinical and endoscopic outcomes while maintaining an acceptable safety profile and is commonly utilized by physicians to treat refractory or severe disease [12,60,61].

Table 2 provides a summary of the key studies that have investigated the role of combination therapy in IBD patients with study design, population, and intervention, along with key findings.

The SONIC trial explored the efficacy of infliximab and azathioprine monotherapies compared to the combination of both drugs; it showed that patients receiving combination therapy achieved significantly higher rates of corticosteroid-free clinical remission and lower rates of immunogenicity [19]. UC-SUCCESS showed similar superiority of the combination of infliximab and azathioprine in patients with UC [57]. A subsequent post hoc analysis of SONIC also showed that, despite the fact that patients on combination therapy achieved higher infliximab concentrations, the higher rates of efficacy seen were driven by higher drug levels and not by the use of combination therapy [13]. The COMMIT trial studied the efficacy of combining infliximab and parenteral methotrexate. Even though it did show that patients receiving the combination of infliximab and methotrexate had achieved higher infliximab levels and lower rates of ADA than those receiving monotherapy, no benefit was found when comparing clinical outcomes [58].

Combination therapy plays an important role in patients who have a higher risk of achieving sub-therapeutic anti-TNF levels and in those with a higher risk of developing immunogenicity. Patients who develop anti-drug antibodies to one anti-TNF are much more likely to develop antibodies to subsequent anti-TNF use [59], and, in this scenario, TDM with combo-therapy can be helpful to prevent immunogenicity to a second anti-TNF. Roblin et al. randomized IBD patients who had a loss of response to the first anti-TNF given as monotherapy and were starting a second anti-TNF to either receive monotherapy or combination therapy. Those who received the combination had a significantly lower risk of developing clinical non-response and developing antibodies to the second agent [62].

An important novel approach when using TDM in the combination therapy of anti-TNF and a thiopurine is to optimize therapy not only by measuring the drug levels of the biologic but also by measuring 6-TGN and optimizing the thiopurine to maximize the pharmacokinetic augmentations of the biologic. This is particularly important, as optimizing thiopurines (via dose adjustment or pharmacologic manipulation) may be significantly more cost-effective than increasing the dose of the biologic. The COMBO-IBD study investigated how the use of thiopurine or methotrexate, and 6-TGN levels were correlated with the achieved infliximab concentrations. Patients with 6-TGN levels ≥ 146 pmol per 8 × 10^8^ RBC or those receiving oral methotrexate had achieved higher drug concentrations than the patients on infliximab monotherapy or those receiving concomitant thiopurine that had 6-TGN levels < 146 pmol per 8 × 10^8^ RBC [63]. Even though the study could only demonstrate an association, it reasonably suggested optimizing thiopurine in patients receiving combination treatment and that those achieving low infliximab concentrations are at a high risk of developing immunogenicity. Interestingly, the same study also showed that this phenomenon is not seen in patients on vedolizumab or ustekinumab, and the use of combination therapy, independent of the agent or 6-TGN concentration, was not associated with higher drug levels of these two biologics [63].

As previously mentioned, it is still unclear whether the better outcomes seen with combination therapy are mainly driven by a pharmacokinetic augmentation or whether there is a synergistic mechanism when combining both drugs [18,19,20,57]. While more studies are warranted, if the positive outcomes are really due to the better pharmacokinetics, it would be interesting to see whether a comparable efficacy can be achieved by using infliximab monotherapy and proactively adjusting dosing without the need for combo-therapy.

## 5. Role of TDM When Restarting Anti-TNFs after a Drug Holiday

Circumstances arise where patients may discontinue anti-TNF and are subsequently restarted after a drug holiday. TDM can be useful in identifying patients who are more likely to develop infusion reactions and non-responses. A practical approach is to perform proactive TDM one to two weeks following re-exposure to anti-TNF therapy; the presence of ADAs suggests a higher likelihood of an infusion reaction occurring with continued dosing and warrants a treatment change [64,65,66]. The use of combination therapy can decrease the risk of developing ADAs in these patients, and while checking thiopurine metabolites soon after restarting therapy may be too early, optimizing the thiopurine after induction aiming to achieve adequate 6-TGN levels is reasonable [64,65,66]. In situations where an adequate level is present without ADAs after anti-TNF re-exposure, proactive TDM may also help to avoid combo-therapy.

Genetics, specifically the HLA-DQA1*05 variant, has been associated with a higher risk of immunogenicity [67]. However, its role in clinical practice is still unclear. In a post hoc analysis of the precision infliximab trial, HLA-DQA1*05 carriage was not associated with ADA formation or higher drug durability [68]. Another single-center retrospective cohort suggests that carriers of this genotypic variant under a proactive TDM strategy are not associated with a higher risk of treatment cessation or worse clinical outcomes [69]. Proactive TDM may be a better predictor of immunogenicity, but larger prospective studies are needed to confirm this.

### TDM and De-Escalation of Combination Therapy

De-escalating treatment in patients receiving combination therapy is another strategy used in select patients, mainly aiming to decrease the risk of potential adverse events. A randomized controlled trial comparing azathioprine/6-mercaptopurine or methotrexate discontinuation vs. continuation in patients receiving a standard dose of infliximab showed that, after 6 months of combination therapy, continuing the immunosuppressant did not offer a clinical benefit. However, the patients who continued the combination therapy had higher infliximab drug levels [70,71]. A study by Sokol et al., however, did find that immunomodulator discontinuation on combo-therapy infliximab led to disease exacerbation, the development of complications, and the need to switch therapies [72]. Overall, it is reasonable to use TDM when considering de-escalation [42]. Roblin et al. demonstrated in an open-label, prospective, randomized trial that a reduction in the dose of thiopurine after 6 months of stable remission (>1 year of treatment) on combo-therapy with infliximab was as effective as full-dose azathioprine, but discontinuation led to a significant drop in the infliximab trough level, even though no major impact on clinical outcomes was observed [73]. In the patients who underwent the thiopurine dose reduction, 6-TGN < 146 pmol per 8 × 10^8^ RBC was associated with worsening infliximab pharmacokinetics. In this setting, it is reasonable to use TDM not only when completely discontinuing the immunosuppressant but also when reducing the azathioprine dose and using 6-TGN levels to determine an optimal threshold level, ultimately aiming to concomitantly increase efficacy and safety.

From a biological standpoint, it would also be sensible to check the drug level before and after de-escalation (or dose reduction). The risk of requiring infliximab dose escalation, drug discontinuation, and IBD surgery is much lower when patients maintain a higher infliximab trough when the immunomodulator is stopped [71]. 

TDM data specific to golimumab use in UC and certolizumab in CD in the setting of combination therapy are lacking. A subgroup analysis of observational data suggests that a lower number of patients develop immunogenicity with these drugs than with infliximab and adalimumab. More data are needed to draw meaningful conclusions regarding TDM, immunogenicity risk, and combo-therapy de-escalation [74,75].

## 6. Discussion

While the combination therapy of anti-TNF and immunomodulators can offer higher effectiveness, a relatively high number of patients still do not respond to treatment or lose response. A large and evolving body of evidence has shown that the use of TDM in patients receiving monotherapy and combination therapy of anti-TNF can be tremendously useful. Since reactive and proactive TDM strategies are also cost-effective, their increased utilization can help treat IBD patients in a more cost-effective way. Another benefit of using a monitored approach includes the avoidance of potential adverse effects seen with combo-therapy.

While the findings in this review have significant implications for clinical practice, unfortunately, the widespread adoption of TDM remains an important barrier. For example, most centers rely on external laboratories, and there might be a significant lag time from sampling and laboratory processing until the clinician receives an actionable result. Furthermore, there is a lack of consensus on optimal target concentrations, which likely vary based on the type of assay used, phenotype of the disease, inflammatory burden, and timing through the dose cycle [76]. The implementation of commercially available point-of-care testing could reduce the lag time, while the standardization of assays to enhance reliability and quality control could help to minimize some of these issues [77,78]. Nevertheless, factors like cost, accessibility, and external validation need careful consideration before recognizing the impact of real-time value on clinical decision making.

As more data become available, personalized TDM strategies and their use in combination with treat-to-target strategies are becoming a standard in IBD management. We might see elements of pharmacogenetics and pharmacokinetic-modeled dashboards aided by artificial intelligence where dosing and monitoring are tailored to each patient [79]. Future studies looking into precision medicine considering multiple patient-centered variables are needed. Furthermore, studies looking into the optimization of newer biologics and small molecules used in the treatment of IBD are also warranted.

## 7. Conclusions

The TDM of anti-TNFs in patients with IBD has been proven to be a useful tool. In patients receiving combination therapy, it can also provide an opportunity to further optimize therapies, especially in patients with non-response to drugs and those not achieving the desired pharmacokinetic effect despite concomitant thiopurine use, and to help guide treatment de-escalation or re-initiation. Conversely, the use of combination therapy and the use of TDM in patients receiving vedolizumab or ustekinumab do not seem to offer a clear benefit.

### Limitations

It is important to acknowledge the limitations inherent in the available literature, as the quality and consistency of reporting across the included studies may vary, potentially affecting the generalizability of the findings. First, overall, there is a limited amount of evidence regarding the use of TDM in combination therapy. Also, our review included articles published in English, which may have omitted relevant studies published in other languages. Second, the heterogeneity of the included studies, in terms of study design, population, and intervention, might have influenced the overall conclusions. The review process may also have inherent biases, such as a potential selection bias in the studies included.

## Figures and Tables

**Table 1:** Select studies comparing proactive vs. reactive TDM.

Study Name/Reference	Study Design	Population	Intervention	Key Findings
TAXIT [28]	Randomized trial	IBD patients on infliximab	Proactive TDM vs. as-needed dose adjustment	Proactive TDM was not superior
TAILORIX [29,30]	Randomized trial	IBD patients on infliximab	Proactive TDM	Results similar to TAXIT
PAILOT [31]	Non-blinded RCT	Pediatric CD patients on adalimumab	Proactive dose adjustment	More efficacious than reactive approach
NOR-DRUM A and B [32,33]	Randomized study	IBD patients on various treatments	Proactive TDM	No benefit during induction but beneficial during maintenance

**Table 2:** Pertinent studies on combination therapy in IBD.

Study Name/Reference	Study Design	Population	Intervention	Key Findings
SONIC [19]	Randomized trial	CD patients on infliximab and azathioprine	Monotherapy vs. combination	Lower immunogenicity, and combination therapy superior
UC-SUCCESS [57]	Randomized trial	UC patients on infliximab and azathioprine	Monotherapy vs. combination	Results similar to SONIC
COMMIT [58]	Randomized trial	CD patients on infliximab and methotrexate	Monotherapy vs. combination	Lower immunogenicity but no significant difference in clinical outcomes
